# Effectiveness of SNAPPS Implementation in the Family Medicine Residency Program in Erbil: A Randomized Controlled Trial

**DOI:** 10.7759/cureus.64897

**Published:** 2024-07-19

**Authors:** Ghaith S Shindala, Ali S Dauod, Nazdar E Alkhateeb

**Affiliations:** 1 Ministry of Health, Ninevah Health Directorate, Mosul, IRQ; 2 Department of Community Medicine, College of Medicine, Hawler Medical University, Erbil, IRQ; 3 Department of Medical Education/Pediatrics, College of Medicine, Hawler Medical University, Erbil, IRQ

**Keywords:** family medicine, snapps, methods of case presentation, medical education, iraq, clinical reasoning

## Abstract

Background and objectives

There is a crucial need to embrace modern methodologies for enhancing medical education in disciplines such as Family Medicine. The study introduces the SNAPPS (Summarize, Narrow, Analyze, Probe, Plan, and Select) model, a six-step mnemonic representing a learner-centered case presentation approach that streamlines fact reporting while encouraging clinical reasoning, aiming to evaluate the effectiveness of the SNAPPS method as compared to the traditional model of case presentation in Family Medicine outpatient clinics and to gain insights into how students and preceptors perceive it.

Methods

A randomized controlled trial was conducted in Family Medicine outpatient clinics in Erbil, Iraq, from March 15, 2023, to August 30, 2023. Using convenience sampling, all Family Medicine board residents (n=30) in Erbil and six preceptors from the Community and Family Medicine department/College of Medicine/Hawler Medical University were randomly assigned into two groups by using the RAND function in Microsoft Excel (Microsoft Corporation, Redmond, Washington, United States). The SNAPPS method was introduced to the SNAPPS group employing approved tools and methods, while no intervention was needed in the control group. Subsequently, 30 cases were presented in each group with a total of 60 case presentations; the case presentations served as the units for data analysis. Feedback and data were gathered after each presentation using validated data recording sheets.

Results

The study showed a significant advantage for the SNAPPS group over the control group in terms of time efficiency, number of basic clinical attributes covered, and justified diagnoses (P value < 0.001). In the SNAPPS group, 90% of the students sought clarification and information, surpassing 30% in the control group (P-value < 0.001). Almost all SNAPPS group students (96.7%) discussed case-related topics, compared to 43.3% in the controls (P-value<0.001). The SNAPPS group received superior overall ratings from both preceptors and students.

Conclusion

The SNAPPS method enhances clinical diagnostic reasoning in Family Medicine outpatient clinics. It is time-efficient and encourages students to articulate uncertainties, pose questions, and identify case-related topics for self-study.

## Introduction

Clinical teaching's primary objectives include evaluating students' clinical reasoning abilities, advancing, and enhancing their development, as well as giving them clinical practice and feedback on their thinking processes [1]. However, while attempting to assist postgraduate residents in improving their clinical reasoning, medical educators experience a variety of difficulties like uncooperative patients, record requirements, increasing tasks, time restraints, and productivity objectives that conflict with teaching time [2].

Most preceptors use the traditional or patient-centered method, where the trainee presents a case in a standardized format; however, to establish a differential diagnosis and a treatment plan, the preceptor may ask focused questions to clarify the history and physical examination findings during or after the presentation and occasionally followed by a brief mini-lecture that infrequently includes feedback [3].

Studies examining the traditional case presentations to preceptors have demonstrated that students predominantly focus on factual information, with infrequent articulation of their clinical reasoning or expression of case-based uncertainties. Additionally, the teaching points are frequently unspecific, not tailored to the learning level of the individual student, and difficult to apply to future cases [3]. Furthermore, the chance to consider one's own method of clinical thinking is seen as one of the most valuable components of the educational experience by both students and preceptors. Researchers have argued that there is a need for the development of time-effective teaching techniques in the clinical setting that give insights into the students' clinical reasoning strategies and doubts while also enabling the preceptor to stay completely involved in the priority areas of patient care [4].

Two models for enhancing clinical thinking ability, the One-Minute Preceptor (OMP) and the SNAPPS (Summarize, Narrow, Analyze, Probe, Plan, and Select) model have been studied well in outpatient settings [5]. By using these teaching models, preceptors can give the trainees a more specific education without consuming more valuable time [6].

The SNAPPS learner-centered model was first introduced by Wolpaw and colleagues in 2003. It's organized into six steps: (i) Summarize briefly the history and findings, (ii) Narrow the differential diagnosis to two or three relevant possibilities, (iii) Analyze the differential diagnosis by comparing and contrasting the possibilities, (iv) Probe the preceptor about uncertainties, difficulties, or alternative approaches, (v) Plan management for the patient’s medical issues, and (vi) Select case-related issues for self-directed learning [7].

The SNAPPS paradigm relies on a learner-teacher continuum which, in the end, should be led by the learner, but may initially require coaching from the preceptor to assist the learner become comfortable and proficient with the processes. This model also depends on the faculty's established expectation that the learner must play a central role and ask questions [5].

In contrast to the hospital setting, the teacher-student time correlation is different in the outpatient setting. It seems reasonable that the trainee, with dedicated educational time, should take on a central role in establishing the structure of the learning interaction. We stand for a new outpatient education model that focuses on the trainee as a comparable, if not more significant part of an effective educational process in the office considering the role changes created by the office setting that result in dedicated learning time for trainees rather than the trainers [7].

The SNAPPS learner-centered model, though applicable at any stage of learning, is particularly advantageous for advanced learners who have recognized specific areas for self-improvement, aiming to promote both diagnostic and clinical reasoning in an interactive manner [8]. Moreover, it necessitates that both teachers and trainees become familiar with its framework, which typically means that it demands a coordinated approach to be implemented, such as an internship or residency program [5].

Family Medicine is a relatively new field in Iraq and the primary care system in the Kurdistan region faces numerous challenges including the quality of medical training provided to students and healthcare professionals and the organization of health services, in addition to the overcrowded healthcare facilities and insufficient time for physicians to deliver quality patient care [9]. Traditional methods used in many Iraqi medical schools do not adequately prepare students for the real-world complexities they will encounter. Consequently, there is a growing need for new educational approaches that provide opportunities for developing competence and gaining practical experience to prepare doctors to deal with these challenges [10]. A learner-centered, six-step SNAPPS paradigm of case presentation alters the learning experience by compressing the reporting of facts while fostering clinical thinking without losing more valuable time [6], which aligns well with these educational needs.

The aim of this study is to evaluate the effectiveness of the SNAPPS model as compared to the traditional model of case presentation in Family Medicine outpatient clinics. The objectives of the study are to assess how well SNAPPS facilitates clinical reasoning and enhances time management in an outpatient setting when compared to the traditional method of case presentation and to gain insights into how the students and professors perceive it. We hypothesize that the SNAPPS method will improve clinical diagnostic reasoning skills and time management in outpatient settings compared to the traditional method.

This article was previously presented as a meeting abstract at the 4th International Conference of the College of Medicine, Hawler Medical University, 2024, on January 11, 2024, in Erbil, Iraq.

## Materials and methods

This was a randomized controlled trial conducted at the outpatient clinics of the Family Medicine teaching health centers in Brayati, Shady, and Azadi primary healthcare centers (PHCs) in Erbil city, Iraq, from March 15, 2023, to August 30, 2023, followed by database analysis and interpretation. The trial was registered at ClinicalTrials.gov (ID: NCT06484010).

Study sample and sample size

Thirty residents and six preceptors from the Department of Community and Family Medicine were selected using the convenience sampling method. These participants were randomly assigned into two groups using the RAND function in Microsoft Excel (Microsoft Corporation, Redmond, Washington, United States): the intervention group, which used the SNAPPS technique, and the control group, which followed the traditional case presentation method.

Participants

Eligible residents were postgraduate students pursuing a board degree in Family Medicine after completing their MBChB (Bachelor of Medicine and Bachelor of Surgery) and internship. This included 16 residents from the Arabic Board of Health Specialties fellowship and 14 residents from the Kurdistan Board of Medical Specialties fellowship. By chance, all residents included in the study were female. Given the limited number of residents, all eligible residents were included in the study.

Eligible preceptors were faculty members at the College of Medicine, Hawler Medical University, who frequently instructed undergraduate and postgraduate students in the family medicine teaching health facilities. This group included two assistant professors and four lecturers, comprising two males and four females.

Case Selection

The cases were routine cases typically encountered in the PHC family medicine clinics, selected according to the patient flow and the residents’ preferences, taking into account the differences in their scientific levels relative to their years of training.

Sample Size

Given the limited number of Family Medicine tutors and postgraduate residents, all available and eligible participants were included in the study. Therefore, no formal power calculation was performed. The total sample size consisted of 30 residents and six preceptors.

Randomization

To minimize selection bias, the 30 residents were randomly assigned to either the SNAPPS group (intervention) or the traditional case presentation group (control). The randomization process was conducted using the RAND function in the Microsoft Excel program. Each resident was assigned a random number generated by the RAND function, and these numbers were then sorted to allocate residents into two groups.

Similarly, the six preceptors were randomly assigned to the two groups using the same RAND function in Excel. Each preceptor was assigned a random number, and the numbers were sorted to ensure an equal distribution of preceptors between the two groups, with three preceptors in each group to maintain consistency in instruction and evaluation.

This methodological approach ensured that all available and eligible residents and preceptors were included, and randomization was conducted in a manner that minimized selection bias. This design maximizes the validity and applicability of the study findings within the constraints of the study setting

**Figure 1:**
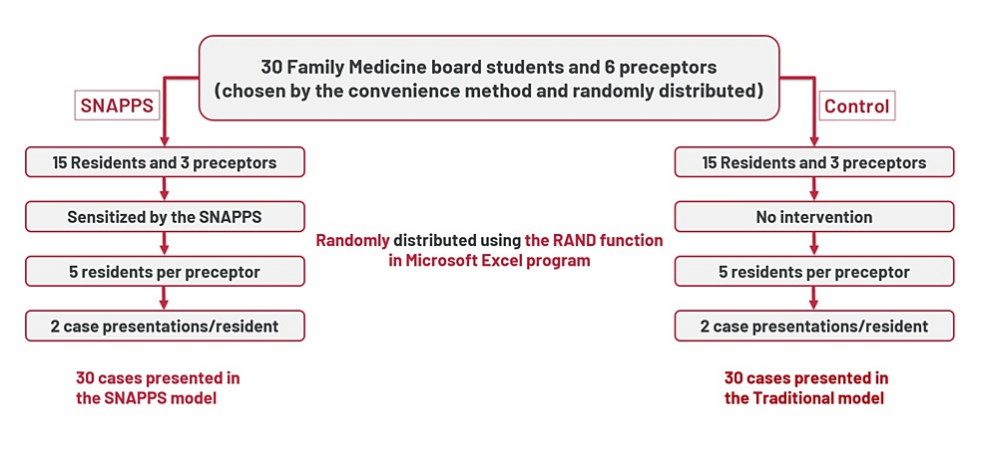
Method of Sampling and Randomization SNAPPS: Summarize, Narrow, Analyze, Probe, Plan and Select

After randomization and assignment of residents and preceptors, each preceptor was responsible for supervising five residents. Each resident was required to prepare and present two different cases according to their preferences during their daily training at the health centers, resulting in a total of 60 case presentations (30 presentations in each group). The case presentations served as the units for data analysis, as illustrated in Figure 1.

Blinding

To prevent contamination between groups, both the students and the preceptors in each group were unaware of the participants (students and preceptors) and the method used for case presentations in the other group. We ensured this by explicitly instructing the participants in both groups, not to discuss any details of the study, including the methods and content of their case presentations, with individuals in the other group. This instruction was emphasized during the initial briefing and reinforced throughout the study period. 

Intervention and study procedures

The intervention was the implementation of the SNAPPS model as a method of case presentation in outpatient settings.

The Experimental Group (SNAPPS Group)

All the preceptors and residents in the SNAPPS group received orientation and training one week before the beginning of presentations and data collection through a training session that was carried out by the authors at Brayati PHC training hall for a duration of more than two hours. The session included a preview of the traditional method, an introduction to the SNAPPS method, role play, and clarification of doubts.

The training materials included a presentation explaining the SNAPPS method in detail with examples [3,7], instructional videos [11,12], and handouts highlighting the six steps of the SNAPPS method.

All the participants had the opportunity to ask questions and clarify their uncertainties. Furthermore, before the beginning of the presentations, by 72 hours, there was a double check by the principal investigator with all participants regarding the method and all doubts were clarified.

The Control Group (Traditional Case Presentation Group)

According to Karen et al., as both learners and preceptors are familiar with the traditional method of case presentation, no special training is required [3].

The traditional method of case presentation, in which the trainee presents the case in a standardized format, is used by the majority of preceptors. In order to establish a differential diagnosis and a treatment plan, the preceptor may ask a series of focused questions to further clarify the history and physical examination findings, during or after the case presentation, and it is occasionally followed by a quick mini-lecture that infrequently incorporates feedback [3].

We emphasized to the control group participants that their presentations should follow the usual structure they are accustomed to, without any modifications or additional training. This ensures that the comparison between the SNAPPS group and the control group accurately reflects the impact of the SNAPPS intervention on case presentation and clinical reasoning skills.

Outcome measures

Primary Outcome Measures

Assessment of SNAPPS outcomes: Various dependable variables were used to assess the outcomes of each case presentation by the preceptors, designed by using appropriate previous research and databases [2,7].

The dependable variables were originally developed by Wolpaw et al. [7]. They include: (i) summarizing patient findings (total, summary, and discussion presentation times), (ii) providing differential diagnosis (number of diagnoses in the differential, number of basic attributes supporting the differential), (iii) analysis of possibilities of differential diagnosis (number of justified diagnoses and comparing contrasting), (iv) expressing uncertainties and obtaining clarification (number of student-initiated questions or discussions about uncertainties or diagnosis), (v) discussing patient management (number of student-initiated questions or discussions about management), (vi) identifying case related reading (number of students discussed case related topics and resources) [7].

The data recording form that included these variables was the same as the one used in a similar study conducted in India by Jain et al. [2], and it was obtained from the corresponding author of that study to be utilized in this study.

Effectiveness of clinical reasoning: The Diagnostic Thinking Inventory (DTI) is a self-report instrument specifically validated to assess clinical reasoning, developed by Bordage and colleagues [13]. This inventory is designed to measure two primary cognitive features: flexibility in thinking and evidence of structure in memory.

The DTI is best utilized as a debriefing tool in relation to one or two specific cases, not as a general measure, in accordance with case specificity [14].

It comprises 41 items that probe various aspects of diagnostic thinking. Each item includes a stem, two accompanying statements, and a rating scale. The rating scale is a continuum between the two statements, with responses ranging from one to six. This scale allows for a nuanced assessment of where the respondent's diagnostic thinking lies between the two extremes presented in each item.

In this study, the DTI questionnaire was given to students in both the SNAPPS and traditional case presentation groups following the final case presentation. This timing ensured that the students' responses reflected their most recent clinical reasoning experiences. The scores were then calculated and analyzed in the categories of thinking flexibility and structure of memory. This allowed for a comparative assessment of how well each teaching method influenced these crucial aspects of diagnostic thinking to provide insights into the effectiveness of the SNAPPS model in enhancing diagnostic reasoning compared to traditional case presentations.

Secondary Outcome Measures

Perception of students and preceptors: A 5-point Likert scale ranging from strongly disagree to strongly agree, was used to take the feedback from the preceptors and residents after each case presentation in both groups.

The components included in the feedback form concisely covered: all aspects of history taking, performed all the steps of general examination, systemic examination findings relevant and in accordance with history, sequencing and formulation of differential diagnosis well organized, the hypothesis of differential diagnosis matched with history and examination, able to speak out all the difficulties faced while case discussion, narration of patient management plan (realistic and appropriate to differential diagnosis), identified sufficient case-based learning issues for self-study, time management during case presentations, and the uniformity and skills of presentation.

In addition, the form included a rating scale ranging from 1 to 10 to enable the participants to provide quantitative feedback.

The components of the feedback forms and DTI questionnaire were the same as the ones used in a similar study conducted in India by Jain et al. [2], obtained from the corresponding author of that study to be utilized in this study.

Ethical consideration

The study protocol was approved by the research ethics committee of Hawler Medical University/ College of Medicine on March 8, 2023 (Meeting code 5, Paper code 5).

All the participants included in this study were informed about the nature and scope of the study and verbal consent was obtained from each of them before participation.

The consent process was witnessed by all the authors prior to participant enrollment in the study. The data were entered into the forms by the participants themselves (preceptors and students). They were required to write their names, the date, and the session number on the form after each presentation. These forms were then handed over to the principal investigator, who was present at and observed all the sessions to ensure accuracy and compliance with the study protocol.

For ethical considerations, after data collection was completed, all residents and preceptors in the control group received SNAPPS orientation using the same materials provided to the SNAPPS group. No further data were collected.

Statistical analysis

Data were entered into tables by using the Microsoft Excel program and then analyzed using the IBM SPSS Statistics for Windows, Version 26.0 (Released 2019; IBM Corp., Armonk, New York, United States). The chi-square test of association was used to compare the proportions of the two groups. Fisher’s exact test was used when the expected frequency (value) was less than five of more than 20% of the cells of the table. An unpaired t-test was used to compare the means of the two study groups. Mann-Whitney test was used to compare the mean ranks of the two groups. A P-value of ≤ 0.05 was considered statistically significant.

## Results

The study included a total of 36 participants, 30 students with six outcome assessors (Preceptors). The demographic details of the study participants are shown in Table 1.

**Table 1:** Demographic details of the participants ^* By chance, all the Family Medicine Board residents in Erbil center were females at the time of the study; **Arab Board of Health Specializations; ***Kurdistan Board of Medical Specialties.^ MBChB: Bachelor of Medicine, Bachelor of Surgery

Students	SNAPPS Group (n=15)	Control Group (n=15)
Age range in years (mean)	27-35 years (29)	28-34 years (31)
Gender*	All females	All females
Educational Level	M.B.Ch.B, Family Medicine Board students (5 from ABHS**, 9 from KBMS***)	M.B.Ch.B, Family Medicine Board students (11 from ABHS**, 5 from KBMS***)
Preceptors	SNAPPS Group (n=3)	Control Group (n=3)
Age range in years (mean)	40-55 years (46.6)	37-43 years (39.6)
Gender	1 male, 2 females	1 male, 2 females
Educational Level	M.B.Ch.B, Family Medicine Board Certified specialists (2 assistant professors and 1 lecturer)	M.B.Ch.B, Family Medicine Board Certified specialists (3 lecturers)

As shown in Table 2, it is evident that the mean and mean rank (7.57 and 37.62, respectively) of the number of basic clinical attributes covered by the SNAPPS method were significantly (p = 0.001) higher than those of the control group (6.63 and 23.38, respectively). All the other indicators were significantly higher in the SNAPPS group than the control group as follows: number of diagnoses kept in differential diagnosis (p < 0.001), number of basic attributes in support of diagnosis in the differential diagnosis (p < 0.001), number of justified diagnosis in the differential diagnosis (p < 0.001), number of distinct comparisons made between two diseases (p < 0.001), number of uncertainties expressed and obtained clarifications (p < 0.001), and overall rating out of 10 (p = 0.003).

**Table 2:** Comparing the numerical dependable variables between the SNAPPS and control groups. ^*By Mann-Whitney test. **The dependable variables questions are as follows:^ ^Q1. Number of basic clinical attributes covered (Out of 9)^ ^Q2. Number of diagnoses kept (Dx) in differential diagnosis (DDx)^ ^Q3. Number of basic attributes in support of Dx in the DDx^ ^Q4. Number of justified Dx in the DDx^ ^Q5. Number of distinct comparisons made between two diseases^ ^Q6. Number of uncertainties expressed and obtained clarifications^ ^Q7. Overall rating out of 10^ SNAPPS: Summarize, Narrow, Analyze, Probe, Plan and Select

SNAPPS Group				Control Group			
Q**	Mean (95% CI)	(SD)	Mean Rank	Mean (95% CI)	(SD)	Mean Rank	P*
Q1.	7.57 (7.07-8.06)	(1.33)	37.62	6.63 (6.30-6.97)	(0.89)	23.38	0.001
Q2.	2.77 (2.61-2.93)	(0.43)	42.63	1.57 (1.31-1.82)	(0.68)	18.37	< 0.001
Q3.	3.10 (2.73-3.47)	(0.99)	40.52	1.93 (1.69-2.17)	(0.64)	20.48	< 0.001
Q4.	2.13 (2.00-2.26)	(0.35)	43.33	1.17 (1.03-1.31)	(0.38)	17.67	< 0.001
Q5.	2.30 (1.84-2.76)	(1.24)	41.60	0.70 (0.42-0.98)	(0.75)	19.40	< 0.001
Q6.	1.60 (1.11-2.09)	(1.30)	40.92	0.33 (0.13-0.54)	(0.55)	20.08	< 0.001
Q7.	8.43 (8.00-8.87)	(1.17)	37.10	7.43 (7.03-7.83)	(1.07)	23.90	0.003

The majority (90%) of the juniors of the SNAPPS group sought clarification and information by asking questions and acknowledging their uncertainties, compared with 30% of the control group (p < 0.001). All the juniors (100%) of the SNAPPS group discussed the patient’s management plan, compared with 70% of the control group (p = 0.005). Almost all (96.7%) of the juniors of the SNAPPS group discussed case-related topics and resources, compared with 43.3% of the control group (p < 0.001).

Regarding the mean of the total presentation time of the SNAPPS group (6.80 minutes), it was significantly less than that (8.65 minutes) of the control group (p < 0.001). The mean duration of discussion in the SNAPPS group (5.20 minutes) was significantly (p = 0.014) more than that of the control group (4.05 minutes), but the mean duration of summary of the SNAPPS group (1.61 minutes) was significantly (p < 0.001) less than that of the control group (4.60 minutes) as shown in Table 3.

**Table 3:** Comparing the other dependable variables between the SNAPPS and the control groups. ^*By Fisher’s exact test. †By unpaired t test.^ SNAPPS: Summarize, Narrow, Analyze, Probe, Plan and Select

Outcome		SNAPPS Group	Control Group	P
Does the student seek clarification and information by asking questions and acknowledging their uncertainties?	Yes (%)	27 (90.0)	9 (30.0)	
	No (%)	3 (10.0)	21 (70.0)	<0.001*
Did the student discuss patient management plan?	Yes (%)	30 (100.0)	22 (73.3)	
	No (%)	0 (0.0)	8 (26.7)	0.005*
Did the student discuss case-related topics and resources?	Yes (%)	29 (96.7)	13 (43.3)	
	No (%)	1 (3.3)	17 (56.7)	<0.001*
Total		30 (100.0)	30 (100.0)	
Total presentation length (minutes)	Mean (95% CI) (SD)	6.80 (6.02-7.57) (2.08)	8.65 (8.12-9.17) (1.41)	<0.001†
Duration of discussion (minutes)	Mean (95% CI) (SD)	5.20 (4.45-5.93) (1.99)	4.05 (3.51-4.59) (1.44)	0.014†
Duration of summary (minutes)	Mean (95% CI) (SD)	1.61 (1.33-1.87) (0.72)	4.60 (4.22-4.97) (1.01)	< 0.001†

Table 4 shows that the mean and mean rank of the first preceptors’ indicator (concisely covered all aspects of history taking) were 4.23 and 34.48, respectively, in the SNAPPS group, compared with 3.83 and 26.52, respectively, of the control group but the difference was close to the level of significance (p = 0.055). The parameters of all the other indicators were significantly higher in the SNAPPS group than in the control group based on the preceptor’s opinion.

**Table 4:** Preceptors’ feedback (evaluation) of junior doctors' presentations in the SNAPPS and control groups (based on Likert scale parameters). ^*By Mann-Whitney test. L** = Likert scale.^ ^L1. Concisely covered all aspects of history taking. ^ ^L2. Performed all the steps of general examination. ^ ^L3. Systemic examination findings were relevant and in accordance with history. ^ ^L4. Sequencing and formulation of differential diagnosis were well organized. ^ ^L5. Hypothesis of differential diagnosis matching with history and examination. ^ ^L6. Able to speak out all the difficulties faced while case discussion. ^ ^L7. Narration of patient management plan – realistic and appropriate to differential diagnosis. ^ ^L8. Identified sufficient case-based learning issues for self-study. ^ ^L9. Time management during case presentations. ^ ^L10. Uniformity and skills of presentation.^ ^L11. Total score preceptors’ feedback.^ SNAPPS: Summarize, Narrow, Analyze, Probe, Plan and Select

SNAPPS Group				Control Group			
L**	Mean (95% CI)	(SD)	Mean Rank	Mean (95% CI)	(SD)	Mean Rank	P*
L1.	4.23 (3.96-4.51)	(0.73)	34.48	3.83 (3.54-4.13)	(0.79)	26.52	0.055
L2.	4.27 (4.03-4.51)	(0.64)	35.25	3.77 (3.45-4.09)	(0.86)	25.75	0.020
L3.	4.03 (3.78-4.28)	(0.67)	35.60	3.60 (3.41-3.79)	(0.50)	25.40	0.010
L4.	4.23 (4.00-4.47)	(0.63)	36.80	3.60 (3.30-3.90)	(0.81)	24.20	0.002
L5.	4.20 (3.95-4.45)	(0.66)	39.00	3.20 (2.85-3.55)	(0.92)	22.00	< 0.001
L6.	4.17 (3.97-4.36)	(0.53)	39.43	2.97 (2.56-3.38)	(1.10)	21.57	< 0.001
L7.	4.13 (3.88-4.39)	(0.68)	37.15	3.37 (3.02-3.71)	(0.93)	23.85	0.001
L8.	4.27 (4.07-4.46)	(0.52)	40.65	3.03 (2.69-3.38)	(0.93)	20.35	< 0.001
L9.	4.20 (3.93-4.47)	(0.71)	37.65	3.43 (3.10-3.77)	(0.90)	23.35	0.001
L10.	4.00 (3.74-4.26)	(0.69)	35.33	3.50 (3.21-3.79)	(0.78)	25.67	0.018
L11.	41.73 (40.23-43.23)	(4.01)	41.75	34.30 (32.37-36.22)	(5.17)	19.25	< 0.001

Nearly the same pattern can be observed in Table 5 regarding the students’ (juniors) feedback, where all the parameters of all the indicators were significantly higher in the SNAPPS group than the control group except for the first one (concisely covered all aspects of history taking) where it is evident that the mean rank of the SNAPPS group (34.35) was higher than that of the control group (26.65), but the difference was near to the level of significance (p = 0.064).

**Table 5:** Students’ feedback in the SNAPPS and control groups (based on Likert scale parameters). ^*By Mann-Whitney test. **L = Likert scale.^ ^L1. Concisely covered all aspects of history taking.^ ^L2. Performed all the steps of general examination.^ ^L3. Systemic examination findings were relevant and in accordance with history.^ ^L4. Sequencing and formulation of differential diagnosis were well organized.^ ^L5. Hypothesis of differential diagnosis matching with history and examination.^ ^L6. Able to speak out all the difficulties faced while case discussion.^ ^L7. Narration of patient management plan – realistic and appropriate to differential diagnosis.^ ^L8. Identified sufficient case-based learning issues for self-study.^ ^L9. Time management during case presentations.^ ^L10. Uniformity and skills of presentation.^ ^L11. Overall rating (out of 10).^ ^L12. Total students feedback score.^ SNAPPS: Summarize, Narrow, Analyze, Probe, Plan and Select

SNAPPS Group				Control Group			
L**	Mean (95% CI)	(SD)	Mean Rank	Mean (95% CI)	(SD)	Mean Rank	P*
L1.	4.13 (3.88-4.39)	(0.68)	34.35	3.77 (3.48-4.06)	(0.77)	26.65	0.064
L2.	4.17 (3.87-4.46)	(0.79)	36.17	3.60 (3.33-3.87)	(0.72)	24.83	0.007
L3.	4.23 (3.98-4.49)	(0.68)	34.63	3.83 (3.54-4.13)	(0.79)	26.37	0.047
L4.	4.43 (4.20-4.67)	(0.63)	39.48	3.47 (3.15-3.79)	(0.86)	21.52	< 0.001
L5.	4.37 (4.12-4.62)	(0.67)	36.78	3.83 (3.61-4.05)	(0.59)	24.22	0.002
L6.	4.67 (4.46-4.87)	(0.55)	40.60	3.43 (3.01-3.86)	(1.14)	20.40	< 0.001
L7.	4.17 (3.92-4.41)	(0.65)	36.25	3.67 (3.42-3.91)	(0.66)	24.75	0.005
L8.	4.57 (4.35-4.78)	(0.57)	40.47	3.20 (2.72-3.68)	(1.30)	20.53	< 0.001
L9.	4.47 (4.28-4.66)	(0.51)	38.67	3.53 (3.17-3.90)	(0.97)	22.33	< 0.001
L10.	4.23 (3.98-4.49)	(0.68)	38.18	3.53 (3.32-3.75)	(0.57)	22.82	< 0.001
L11.	8.53 (8.14-8.92)	(1.04)	37.10	7.60 (7.20-8.00)	(1.07)	23.90	0.002
L12.	43.43 (41.98-44.88)	(3.88)	41.48	35.87 (33.61-38.11)	(6.03)	19.52	< 0.001

It is evident in Table 6 that 60% of the juniors of each group got flexibility in thinking scores that were lower than their peers’ mean. The mean rank of this score in the SNAPPS group (16.20) was higher than that (14.80) of the control group, but the difference was not significant (p = 0.683). Regarding the structure of memory scores, the table shows that 33.3% of the SNAPPS group vs. 53.3% of the control group got scores that were lower than the mean of their peers (p = 0.269). No significant difference (p = 0.345) in the mean rank was detected between the two groups.

**Table 6:** Parameters of Diagnostic Thinking Inventory (DTI) of the SNAPPS and control groups. ^*By Chi square test. **By Mann-Whitney test.^ SNAPPS: Summarize, Narrow, Analyze, Probe, Plan and Select

DTI scores	SNAPPS Group	Control Group	P Value
	No. (%)	No. (%)	
Flexibility in thinking			
Lower than their peers' mean (< 85.6)	9 (60.0)	9 (60.0)	1.000*
Higher than their peers' mean (> 85.6)	6 (40.0)	6 (40.0)	
Mean (95% CI) (SD)	84.73 (79.70-89.76) (9.08)	82.87 (78.51-87.22) (7.86)	0.683**
Median	84.0	83.0	
Mean rank	16.20	14.80	
Structure of memory			
Lower than their peers' mean (< 82.8)	5 (33.3)	8 (53.3)	0.269*
Higher than their peers' mean (> 82.8)	10 (66.7)	7 (46.7)	
Mean (95% CI) (SD)	84.0 (77.61-90.39) (11.53)	81.67 (76.42-86.91) (9.47)	0.345**
Median	86.0	82.0	
Mean rank	17.03	13.97	

## Discussion

Using the SNAPPS learner-centered technique for case presentations enabled clinical diagnostic reasoning and case-based uncertainties to be expressed in a busy outpatient setting without prolonging the usual student case presentation time [2]. The fact that this approach is learner-initiated, learner-directed, and learner-centered is what gives it its strengths, and it is more appropriate for the advanced learner, according to several medical educators [3].

Assessment of SNAPPS outcomes

Summarizing Patient Findings

During case presentations, the mean of total presentation time of the SNAPPS group was significantly less than that of the control group by around 1.5 minutes. Interestingly the mean of discussion duration was significantly more than the control group by 1.15 minutes. Additionally, the duration of summary in the SNAPPS group was significantly less than that of the control group by about three minutes, while the mean of the number of basic clinical attributes covered was significantly higher than the control group by almost one.

Compared to a similar study conducted in India [2], our data show similar trends in terms of summary thoroughness, discussion, and summary duration, However, our study revealed that the mean total presentation time in the SNAPPS group was shorter than in the control group, whereas the Indian study found it to be longer by 1.63 minute. The best explanation for this difference lies in the nature of traditional case presentations.

Studies on traditional case presentations to preceptors have revealed that students focus mainly on factual data and infrequently convey their clinical reasoning or case-based doubts [7]; consequently, during the traditional case presentations, the preceptors were constantly asking questions, explaining their point of view and guiding the student during the summary time, this interaction led to a significantly longer duration of summary by around three minutes, what in turn made the total presentation time longer than that of the SNAPPS group by one and a half minute.

Preceptors who need to balance teaching with effective patient care can anticipate that SNAPPS presentations are less time-consuming, and meanwhile, the summary thoroughness is better than the traditional method of case presentation.

Narrowing the Differential Diagnosis

The mean number of diagnoses included in differential diagnosis provided by the SNAPPS group was significantly (P < 0.001) higher than those of the control group. This indicates that the SNAPPS model encourages students to consider a broader range of differential diagnoses.

Our findings align with a similar study conducted in India by Jain et al. [15]. Their study also reported that students using structured approaches like SNAPPS generated more comprehensive differential diagnoses as compared to those using traditional methods. Students in the SNAPPS group were trained to provide two or three differential diagnoses, whereas students in the control group frequently proceeded to the next step right after summarizing the patient’s findings.

Clinical educators cannot effectively evaluate learners’ diagnostic reasoning without understanding their diagnostic hypotheses [4]. By encouraging students to articulate multiple differential diagnoses, the SNAPPS method provides educators with better insight into the students’ thought processes, allowing for more targeted feedback and instruction.

Analyzing the Differential Diagnosis

The students in the SNAPPS group demonstrated significantly superior performance in analyzing their differential diagnoses compared to the control group. This was evident in several key areas as they articulated a higher number of justified diagnoses out loud, made nearly twice as many distinct comparisons between diseases, and covered more basic clinical attributes to support their diagnoses.

These findings are consistent with previous studies from the United States and India [4,16], which have also demonstrated that the SNAPPS approach significantly enhances students’ analytical and justificatory capabilities in differential diagnosis.

To prevent early conclusions and incorrect diagnoses, researchers suggested that learners who rely on nonanalytic reasoning strategies, such as pattern recognition, still need to carry out an analytical confirmation, moreover, analytical techniques, such as comparing and contrasting diagnostic alternatives provide supporting evidence for the confirmation of the initial diagnosis [4].

Probe the Preceptor by Asking Questions About Uncertainties, Difficulties, or Alternative Approaches

Our study’s findings underscore the efficacy of the SNAPPS model in facilitating active engagement among medical students. Within the SNAPPS group, a remarkable 90% of students sought clarification and information by asking questions and acknowledging their uncertainties, compared to only 30% in the control group.

These findings align with those of other studies. For example, a study conducted in India [16], which applied the SNAPPS model in pediatric outpatient education, similarly reported a substantial increase in the frequency of student questions and acknowledgments of uncertainty in the SNAPPS group as compared to the control. Moreover, a similar study by Wolpaw et al. [17], demonstrated that students utilizing the SNAPPS model articulated nearly twice as many questions as those in the comparison group. The consistency of these findings across different studies reinforces the effectiveness and generalizability of the SNAPPS model in enhancing active learning across diverse clinical settings.

A notable strength of the SNAPPS model lies in its ability to empower students to direct the content of their learning based on the uncertainties they express during case presentations. This approach shifts the traditional teacher-centered dynamic to a more student-centered paradigm, where preceptors are prepared to teach in accordance with the specific needs of the students [17].

*Plan Management*

The percentage of students who initiated a patient management plan in the SNAPPS group was higher than the control group by 26.7 percentage points, with all residents in the SNAPPS group discussing patient management compared to 73.3% in the control group (P value=0.005). These findings are consistent with previous research conducted in the United States by Wolpaw et al. [4], and in India by Jain et al. and Kapoor et al. [2,16]. The alignment of our results with those from established studies validates the effectiveness of the SNAPPS approach across diverse educational settings and cultural contexts. 

In addition, Initiating the discussion of patient management plans enables the teacher to determine the student's proficiency level and subsequent management strategy [13]. Furthermore, the inclusion of a patient management plan that is realistic and appropriate to the differential diagnosis is essential. Firstly, a management plan that aligns with the actual clinical situation is crucial for ensuring relevant and effective interventions, thereby improving patient outcomes and adhering to best clinical practices [7]. Secondly, directly linking the management plan to the differential diagnosis highlights the student's ability to integrate diagnostic reasoning with appropriate treatment strategies, demonstrating a deeper understanding of the clinical process and underscoring the importance of accurate diagnosis in patient care [7].

Select Case-Related Topics for Self-Study

The selection of case-related topics and resources for self-study occurred in almost all (96.7%) of the students of the SNAPPS group compared with 43.3% of the control group.

Jain et al. [2], Wolpaw et al. [4], Kapoor et al. [16], and Sawanyawisuth et al. [18] all demonstrated that the SNAPPS technique significantly facilitates the selection of case-related topics for self-learning. These studies collectively support our findings, indicating that the SNAPPS method effectively promotes self-directed learning among medical students.

This finding is important because as Bowen [1] emphasized, preceptors should promote good reading habits, particularly because readings related to the learners' patients help reinforce their memory on both cognitive and experiential levels.

Effectiveness of clinical reasoning

The mean scores for thinking flexibility were comparable between the SNAPPS group (84.73) and the control group (82.87). Similarly, the structure of memory scores was 84.0 in the SNAPPS group and 81.67 in the control group. Although the SNAPPS group showed slightly higher scores in both areas, the differences were not statistically significant. Our findings align with the results of Jain et al., who also reported no significant difference in cognitive flexibility and memory structure between the SNAPPS group and the traditional case presentation group [2].

The DTI is best utilized as a debriefing tool in relation to one or two specific cases, not as a general measure, in accordance with case specificity [14].

A clinician's diagnostic accuracy cannot be determined by a single approach alone. Instead, employing a variety of methods is essential to obtain a precise evaluation [19].

The lack of significant differences in thinking flexibility and memory structure scores suggests that while SNAPPS improves certain aspects of case presentation and analysis, it may not have a broad impact on all cognitive domains. This underscores the importance of using a variety of educational tools and methods to address different facets of clinical reasoning and learning.

Perception of preceptors and students

According to the preceptors’ and students’ opinions, the students in the SNAPPS group performed significantly better than those in the control group in various aspects, as they were more systematic in their examinations, their differential diagnoses better aligned with patient history and examination, and they included more differential diagnoses.

These findings align with previous research by Wolpaw et al. [4], which suggests that structured case presentation methods like SNAPPS enhance clinical reasoning and critical thinking skills. This structured approach allows students to systematically analyze patient cases, fostering a deeper understanding and better retention of clinical knowledge.

Additionally, students in the SNAPPS group were more capable of articulating difficulties, their management plans were more realistic and appropriate to the differential diagnosis, they identified more sufficient case-based learning issues for self-study, and they had better time management. The overall rating of the SNAPPS group was also significantly higher than that of the control group, according to both preceptors and students.

The significant improvements observed in the SNAPPS group are consistent with the findings of a similar study in India, which found that the SNAPPS model not only improved students' clinical reasoning but also enhanced self-directed learning, suggesting a positive impact on their overall educational experience [15].

Both preceptors and students agreed that concise coverage of all aspects of history taking in the SNAPPS group presentations was higher than the control group, but the difference was not significant for both groups (p = 0.055 for preceptors and p = 0.064 for students).

This suggests that while SNAPPS improves many aspects of case presentations, it might not significantly enhance the comprehensiveness of history taking compared to traditional methods. The emphasis of SNAPPS on concise, focused presentations according to Bowen [1], could explain this observation. This finding highlights a potential area for further refinement of the SNAPPS method to ensure comprehensive history-taking without compromising conciseness.

Limitations of the study

The study is constrained by several factors. Firstly, the SNAPPS technique being relatively new limits the number of available studies, and our focus was primarily on how students expressed their clinical reasoning and uncertainties.

Additionally, we did not conduct a formal sample size calculation, opting instead to include all 30 Family Medicine residents available at the time of the study, which may have impacted our ability to detect smaller group differences. Although we employed rigorous randomization and statistical methods, the small sample size restricts the generalizability of findings to broader student populations or different medical specialties.

Another limitation is the potential gender bias, as all students involved in the SNAPPS study were by chance female, while the preceptors consisted of two males and four females. This gender disparity may introduce bias and affect the generalizability of the results. Furthermore, we did not conduct a pilot study prior to the main study. Although the questions and variables were adapted from validated previous research and obtained with appropriate permissions, a pilot study could have helped identify any unforeseen issues in the study design and data collection process.

Despite these limitations, our study adhered to ethical standards and rigorous methodology. By comparing SNAPPS with traditional case presentations, we provided valuable insights into its effectiveness in enhancing clinical reasoning among residents. Moving forward, future research should address these constraints to further validate SNAPPS’ broader applicability and effectiveness in medical education settings.

## Conclusions

The SNAPPS method of case presentation enhances clinical diagnostic reasoning in Family Medicine outpatient clinics during case presentations to the preceptors. It is not only time-efficient but also promotes students to articulate uncertainties, pose questions, and identify case-related topics for self-study, thereby fostering deeper engagement in the learning process.

The superior ratings from both preceptors and residents highlight the model’s effectiveness and acceptability in clinical education. The SNAPPS method’s ability to enhance clinical reasoning skills, improve the quality of case presentations, and promote active learning underscores its value as a tool for medical education and suggests its potential for wider implementation in medical training programs.
